# A Light-Thin Chitosan Nanofiber Separator for High-Performance Lithium-Ion Batteries

**DOI:** 10.3390/polym15183654

**Published:** 2023-09-05

**Authors:** Yanghui Song, Guanglei Zhao, Sihan Zhang, Chong Xie, Xiaofeng Li

**Affiliations:** 1 State Key Lab of Pulp and Paper Engineering, School of Light Industry and Engineering, South China University of Technology, Guangzhou 510641, China; 2 School of Food Science and Engineering, South China University of Technology, Guangzhou 510644, China

**Keywords:** chitosan, nanofiber, separator, lithium-ion battery, nano-porous structure

## Abstract

With the development of portable devices and wearable devices, there is a higher demand for high-energy density and light lithium-ion batteries (LIBs). The separator is a significant component directly affecting the performance of LIBs. In this paper, a thin and porous chitosan nanofiber separator was successfully fabricated using the simple ethanol displacement method. The thickness of the CME15 separator was about half that of mainstream commercial Celgard2325 separators. Owing to its inherent polarity and high porosity, the obtained CME15 separator achieved a small contact angle (18°) and excellent electrolyte wettability (324% uptake). The CME15 separator could maintain excellent thermal dimensional stability at 160 °C. Furthermore, the CME15 separator-based LIBs exhibited excellent cycling performance after 100 cycles (117 mAh g^−1^ at 1 C). The present work offers a perspective on applying a chitosan nanofiber separator in light and high-performance lithium-ion batteries (LIBs).

## 1. Introduction

Lithium-ion batteries (LIBs) are extensively utilized in energy storage systems and portable electronics owing to their high-energy density, high average output voltage, and excellent cycle stability [1,2]. As an inactive component in the LIBs, the separator serves the purpose of isolating the cathode and anode while providing micro-porous pathways to fabricate the migration of ions during the charging and discharging process, but it does not contribute to energy storage [3,4]. Superb separators should process low thickness, high porosity, and robust mechanical strength as these contribute to reducing internal resistance and increasing the ionic conductivity of LIBs [5]. Currently, commercial polyolefin separators, such as polyethylene (PE) and propylene (PP), exhibit excellent electrochemical stability and tensile strength [6,7].

The demand for higher energy storage systems necessitates the development of high-energy density LIBs [8,9,10]. According to the energy density equation, increasing the proportion of active materials by decreasing inactive material is an effective strategy to improve the capacity of LIBs [11]. Previous approaches, such as increasing the thickness of active materials, reducing the weight of current collectors, and enhancing battery group efficiency, successfully boosted the energy density of batteries [12,13,14]. Generally, enhancing the energy density of LIBs involves increasing the loading of active materials and optimizing the volume and weight fractions of inactive components [15]. Unfortunately, relatively little effort has been devoted to systematically investigating the effect of separator thickness on battery energy density. Commercial separators restricted to between 20 and 25 μm can never meet the requirements of high-energy density due to the dry and wet process. These relatively thick separators reduce the available space for containing active components inside the LIBs, thereby hindering further enhancement of the high capacity performance of LIBs [16]. Additionally, low porosity caused by greater thickness and inherent hydrophobicity leads to poor electrolyte uptake. Moreover, these separators are non-degradable materials, posing significant environmental issues [17]. 

Thickness is one of the important parameters used for measuring the electrochemical and safety performances of separators. Due to the demand for high-energy-density LIBs, there is a trend to pursue thinner and lighter separators [18]. Reducing the separator thickness can increase the porosity of the separator and also shorten the migration path of ions, thus decreasing the internal resistance [19]. Horvath et al. [20] reported a semi-empirical equation which showed that energy density was lightly improved by adjusting the separator volume. Zhong et al. [21] summarized the effect of separator thickness on lithium-based battery energy density and found that there is a significant increase in volumetric energy density as the internal resistance is reduced. Moreover, reducing the separator thickness from 10 μm to 5 μm yielded a greater increase in energy density than when reduced from 15 μm to 10 μm [22]. Zhu et al. [23] reported that the energy density of graphite (Gr)/NCM523 batteries was effectively increased by 11.5% when the thickness of the separator decreased from 25 μm to 7 μm. Undoubtedly, optimizing the thickness of the separator is of great significance to increasing battery energy density [24]. However, thinner polyolefin separators result in a significant reduction in mechanical strength, thereby raising the safety risk of LIBs. In brief, thin separators exhibit three factors that affect the performances of LIBs; (1) decreasing the internal resistance improves the energy density; (2) saving the materials reduces cost; (3) reducing the separator thickness will raise safety risk. Therefore, a good balance between ionic conductivity and mechanical strength needs to be maintained.

To address these issues, there is increasing research on alternatives to commercial polyolefin separators with sustainable materials such as cellulose [25], chitin [26], poly(L-lactic acid) [27], and silk [28]. Natural biomass nanofiber-based separators have emerged as promising choices due to their high electrolyte affinity, excellent thermal stability, robust mechanical strength, environment-friendly properties, and low cost [29]. It is important that natural biomass materials have demonstrated potential in balancing separator thickness and mechanical strength. Zhang et al. [30] successfully fabricated a cyanoethyl-chitin nanofiber-based porous separator (CCM) with a thickness of 12 μm and a mechanical strength of 120 MPa. The energy density of batteries assembled with a CCM-3 separator was comparable to those using a traditional PP separator. Furthermore, Pan et al. [31] demonstrated that the thickness and porosity of biomass fiber-based separators can be adjusted easily by controlling the number of materials used.

However, the presence of biomass nanofibers with a high abundance of polarity functional groups (-OH), such as nanocellulose, can lead to membranes with poor porosity and a very small pore size, which is a result of the intense hydrogen bonding between nanofibers, which hinders the Li^+^ migration in the separators [32]. To improve Li^+^ transport in natural biomass nanofiber-based separators, the porosity and pore structure can be adjusted by using a pore former during the formation. However, the reported pore formers such as nano-silica particles may result in an increase in the weight of the separator [33]. To address this issue, researchers tend to change the diameters of the fibers to adjust the porosity and pore size of the separators. Wang et al. [34] increased the beating revolutions of 2,2,6,6-tetramethylpiperidine-1-oxyl (TEMPO) oxidation of cellulose to enhance the performances of a separator. However, although the prepared separators exhibited good porosity, their mechanical strength unexpectedly decreased [35]. Additionally, surface chemical modification methods have been used to induce a steric hindrance effect among nanofibers and prevent the flocculation of nanofibers. Unfortunately, those methods tend to increase the thickness of the separator [36]. Chitosan, as the second most abundant natural degradable material, is a cationic alkaline polysaccharide derived from deacetylated chitin and has a large number of amino groups, which can facilitate strong interactions with Li^+^ [37]. Moreover, the high crystallinity and dense hydrogen chain network between the fibers allow the chitosan separators to contribute to high mechanical strength [38,39].

**Figure 1:**
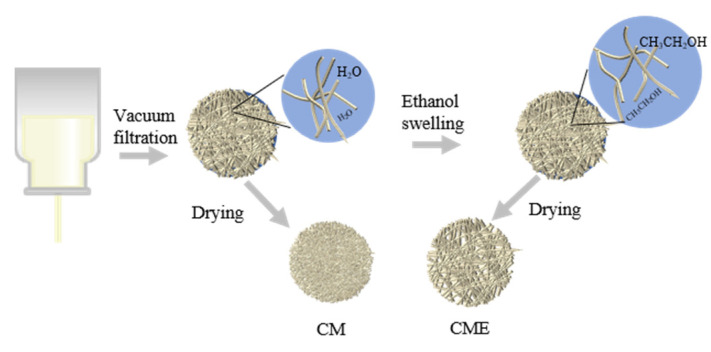
Schematic illustration of the fabrication of chitosan-based membranes.

In this work, we explore the use of degradable and renewable chitosan materials as an alternative to polyolefin separators in LIBs. We successfully developed a light and thin chitosan nanofiber separator by using a mature paper-making process. Subsequently, the nanofiber membranes were immersed in an ethanol bath to create porous chitosan nanofibers separators for LIBs (Figure 1). After drying, the ethanol evaporated without residue. The results demonstrate that the ethanol bath can significantly improve the porosity and pore size of a separator. The thickness of the as-presented CME15 separator was about half that of Celgard separators. Moreover, the obtained separators not only exhibited high porosity, superior thermal stability, and excellent electrolyte wettability, but also demonstrated better electrochemical performances compared to commercial Celgard separators. This work provides a viable option for producing thin, light and degradable separators for LIBs.

## 2. Experiments

### 2.1. Materials

Regenerated chitosan fiber (CSF, ≥95% deacetylated ratio, viscosity ≥1000 mPa·s) was purchased from Shandong Laizhou Highly Bio-chemicals Co., Ltd., (Laizhou, China). Liquid electrolyte, 1 mol L^−1^ lithium hexafluorophosphate (LiPF_6_) in mixed solution (ethylene carbonate/dimethyl carbonate/diethyl carbonate (EC/DMC/DEC) = 1/1/1, *v*/*v*/*v*) was purchased from Shenzhen Kejing Co., (Shenzhen, China). Lithium iron phosphate (LiFePO_4_), acetylene black, polyvinylidene difluoride (PVDF), and 1-methyl-2-pyrrolidinone (NMP) were obtained from Aladdin Shanghai Co., Ltd., Shanghai, China. The polypropylene/polyethylene/polypropylene (PP/PE/PP, Celgard2325) separator was purchased from Shenzhen Kejing Co., (Shenzhen, China) for comparison.

### 2.2. Preparation of the Chitosan Nanofibers Separator

Chitosan fibers were cut into 5 mm long pieces by a cutting mill (CM200, Grinder, Beijing, China) and swollen with deionized (DI) water for 8 h. The solid content of the chitosan slurry was maintained at 10% (*w*/*w*). The chitosan fiber slurry was processed by a PFI refiner (Hamjern Maskin 621, AS, Olso, Norway) and beaten under a pressure of 3.33 N/mm with 60,000 revolutions. The obtained chitosan fiber slurry was diluted to 0.5 wt% with DI water before further treatment. The chitosan fiber slurry was then treated using a microflow nano-homogenizer (Nano DeBEE Next Generation Homogenizers, BEE, Norwood, MA, USA). Under a high pressure of 60 bar, chitosan nanofibers were fabricated using nozzle diameters of 200 μm (D8). To enhance the length-to-diameter ratio, the chitosan slurry was homogenized five times. Finally, the chitosan fiber membranes were prepared by vacuum filtration (0.8 μm, Xingya, Shanghai, China), and following that, the wet membranes were placed into anhydrous ethanol for 12 h. The wet membranes were subsequently dried in a vacuum oven at 60 °C for 12 h. The ethanol-treated chitosan nanofiber membranes were named CME15 (15 μm) and CME25 (25 μm), differentiated by thickness. The CM25 membrane (25 μm) was not treated with ethanol.

### 2.3. Characterization

#### 2.3.1. Morphology of Separators Analyzed by SEM

The samples were mounted onto a sample holder using double-sided conductive tapes and were then coated with a thin Pt layer created by an EMS 300T sputter coater (Quorum Technologies, TT Electronics, Lewes, UK) to minimize charge accumulation during imaging. Scanning electron microscopy (SEM; Quanta FEG 250, FEI, Hillsboro, OR, USA) was used at a voltage of 5 kV to characterize the surface morphologies and fiber diameters.

#### 2.3.2. Porosity

The porosity of the separators was measured by submerging them in an n-butyl alcohol bath for 1 h. The porosity was calculated using Equation (1):

(1) $$\mathrm{Porosity}=\frac{m_{a}/\rho_{a}}{m_{a}/\rho_{a}+m_{b}/\rho_{b}}\times \;100\%$$

where *m_a_* and *m_b_* are the mass of n-butyl alcohol and the separator, respectively, and *ρ_a_* and *ρ_b_* are the density of n-butyl alcohol and the separator, respectively.

#### 2.3.3. Mechanical Property

A universal tensile tester (Instron 3300, Instron, Norwood, MA, USA) was used to assess the stress–strain characteristics of the separators at a speed of 10 mm min^−1^; the width and length of the samples were 10 mm × 30 mm.

#### 2.3.4. Wettability

A contact angle analyzer (DataPhysics, Filderstadt, Germany) was used to evaluate the contact angles (CAs) by dropping 10 μL electrolyte onto the surface of the separator. The electrolyte uptake was calculated using Equation (2):

(2) $$\mathrm{Uptake}\;(\%)\;=[{(m}_{1}-m_{0})/m_{0}]\times \;100\%$$

where *m*_0_ and *m*_1_ are the weights of the separator before and after soaking in the liquid electrolyte, respectively.

#### 2.3.5. Thermal Properties

Differential scanning calorimetry (DSC; NETZSCH TG209F3, Selb, Germany) was used to test the melting peak at a heating rate of 10 °C min^−1^ from 50 °C to 600 °C in a nitrogen atmosphere. An oven was used to measure the thermal-dimensional stability of the separators from 30 °C to 160 °C for 0.5 h. The thermal shrinkage rate was calculated using Equation (3):

(3) $$\mathrm{Thermal}\;\mathrm{shrinkage}\;(\%)=\left[{(s}_{1}-s_{0})/s_{0}\right]\times 100\%$$

where *s*_1_ and *s*_0_ are the areas of the separators before and after heating, respectively.

#### 2.3.6. Electrochemical Characterizations

An electrochemical workstation (CHI 760D, Chenhua, Shanghai, China) was used to measure the electrochemical impedance spectroscopy (EIS). The ionic conductivity of separators was determined through EIS using two stainless-steel (SS) blocking electrodes with a voltage amplitude of 10 mV across a frequency range of 0.1–10^5^ Hz, following the respective equation, and the interfacial resistances of the separators between two lithium plates were also measured. The ionic conductivity was calculated using Equation (4):

(4) $$\sigma =L/R_{b}\times S$$

where σ is the ionic conductivity of the separator, *L* is the thickness of the separator, *S* represents the contact area of the stainless-steel blocking electrode, and R_b_ is the bulk electrolyte resistance as indicated by the Nyquist plot.

The interfacial resistance of Li/Li cells was also measured using EIS. The electrochemical stability of separators was determined using LSV. The working electrode was stainless steel, and the counter electrode was lithium metal. The potential range was between 2.5 V and 5.5 V under a scan rate of 1.0 mV s^−1^ at room temperature [40].

The lithium-ion transport number was measured by combining the chronoamperometry (potentiostatic polarization method) and the EIS, and was calculated using the following Equation (5):

(5) $$t_{\mathrm{Li}+}=\frac{I_{ss}(\Delta V-I_{0}R_{0})}{I_{0}(\Delta V-I_{ss}R_{ss})}$$

where t_+_ is the lithium-ion transference number, *I*_0_ and *I_ss_* are the initial and steady-state current, respectively; *R*_0_ and *R_ss_* are the initial interfacial and steady-state resistance, respectively; Δ*V* is the step potential difference (10 mV).

LiFePO_4_ cathodes (LiFePO_4_/carbon black/PVDF = 8/1/1, *w*/*w*/*w*) were prepared using a doctor-blading process and dried under vacuum at 120 °C for 12 h. A half-battery (2032-type coin) was assembled by sandwiching separators between a LiFePO_4_ cathode and a natural graphite anode and filling with 20 μL of the 1 M LiPF_6_ in EC/DMC/DMC (1:1:1, *v*/*v*) electrolyte. All the cells were placed in an argon-filled glovebox to ensure safety and electrical performance.

A NEWARE battery testing system (CT-4008) was used to evaluate the C-rate capability and cycle performance of the batteries at room temperature. The discharge current densities ranged from 0.1 to 2.0 C under a voltage from 2.5 V to 4.2 V. The cycle performance of batteries was measured at a charge/discharge current density of 1 C/1 C.

## 3. Results and Discussion

The important physical performances of different separators are summarized in Table 1. Thickness is a significant factor used to evaluate the porosities of the separator. Reducing the separator thickness can reduce the internal resistance of the assembled LIBs, which necessitates the preparation of light-thin and high-performance LIBs [31]. The thickness of the CME15 separator was just 15 μm, which was about half that of the Celgard separator, and the CME15 separator presented the highest porosity and electrolyte uptake. The improved battery performances were attributed to the high electrolyte uptake of CME15. Furthermore, the density of CME15 (0.54 g cm^−3^) decreased to 59% of CM25’s density, which is similar to Celgard’s, and this reduction was ascribed to the improved porosity due to the swelling of the ethanol.

### 3.1. Morphology of the New Chitosan Nanofiber Separators

**Figure 2:**
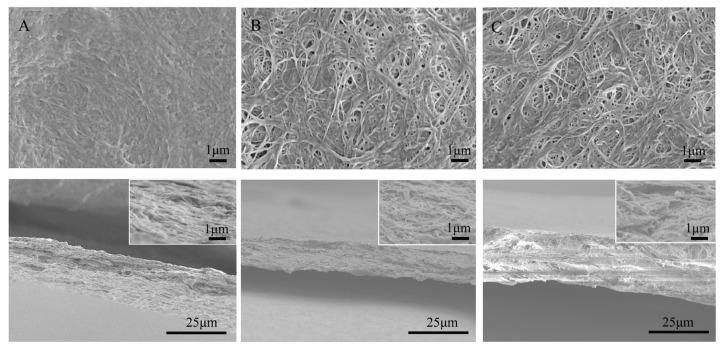
SEM morphology of various separators: (**A**) CM25, (**B**) CME15, (**C**) CME25 and the corresponding cross-sectional images with an illustration of high resolution.

The ethanol bath is a key process in the preparation of porous chitosan nanofiber separators. During soaking, ethanol replaces the water between the chitosan fibers, adjusting the fiber distribution. The microstructures of chitosan nanofiber separators are shown in Figure 2A–C. The ethanol-treated chitosan nanofiber separators (CME15, CME25) were porous structures and had numerous nanoscale pores uniformly distributed on the surface and this disordered distribution of nanofibers in the CME separators can result in an increase in porosity. This is because the addition of ethanol with lower surface tension can improve the spacing between chitosan fibers, making the fiber distribution looser. In contrast, almost no pores existed on the surface of the CM25 separator, this dense structure performance was because the chitosan nanofibers were tightly interwoven due to intensive hydrogen bonding among the nanofibers. Furthermore, compared with the cross-section of the CM25 separator, it can be observed that the CME separators were looser, with 3D interconnected nanopore structures, which were conducive to absorbing more electrolyte, thus improving liquid retention. The Celgard separator prepared via uniaxial stretching technology exhibited narrow pores (Figure S1).

**Figure 3:**
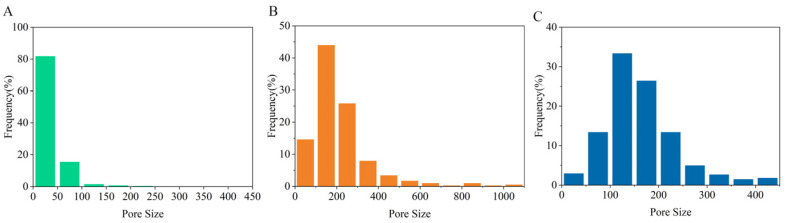
Pore diameter distributions of different separators: (**A**) CM25, (**B**) CME15, and (**C**) CME25.

The pore diameter of the chitosan nanofiber separator was correlated with the stacking density of fibers. The pore diameter of the CM25 separator was mostly less than 50 nm (Figure 3A), whereas the pore diameter of the CME15 and CME25 separators was mainly concentrated in the range of 100–200 nm (Figure 3B,C). In the process of practical application, a separator with rich pore structures provides more migration channels for Li^+^ at the same time, and the uniform pore distribution can also effectively reduce local current in the battery, which inhibits the growth of lithium dendrites [41,42]. In addition, the nano-scale pore channels formed by the interweaving of nanofibers can effectively prevent active substances in the separator, which is conducive to reducing the risk of short circuit [43].

### 3.2. Mechanical Property

**Figure 4:**
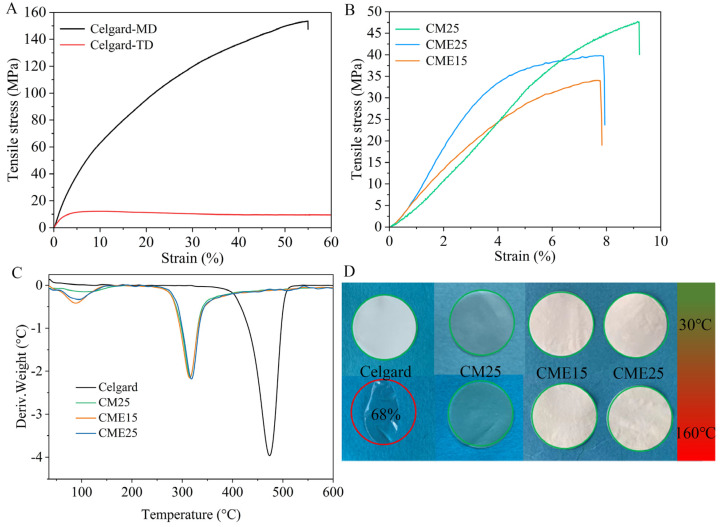
(**A**) Stress-strain curves of the Celgard separator. MD: longitudinal tensile strength; TD: transverse tensile strength. (**B**) Stress-strain curves of a series of chitosan separators. (**C**) DSC of the Celgard and chitosan separators. (**D**) Thermal shrinkage of the Celgard and chitosan separators.

Mechanical strength and thickness are two important parameters used to evaluate the separator performance of LIBs [44]. On the one hand, the separator as a physical barrier isolates the anodes and cathodes of LIBs and mechanically inhibits the growth of lithium dendrites during charging/discharging cycling. On the other hand, in a limited space volume of LIBs, a thinner separator is beneficial to reduce the internal resistance of LIBs. However, the thickness directly affects the mechanical stability of the separator, and it is also necessary to meet the mechanical strength requirements of LIBs. The stress-strain curves of the Celgard separator have a large difference in the transverse and longitudinal directions due to the preparation process, with a transverse tensile strength of 11 MPa and a longitudinal tensile strength of 153 MPa (Figure 4A). The tensile strength of CM25, CME15, and CME25 was 47 MPa, 32 MPa, and 37 MPa, respectively (Figure 4B). The mechanical strength of the chitosan nanofiber separators was less than the longitudinal tensile strength of the Celgard separator but higher than its transverse tensile strength. Furthermore, the tensile strength of CME separators was lower than that of the CM25 separator, owing to the looser structure of CME. In addition, the chitosan nanofibers were randomly dispersed during the preparation process, and thus the tensile strength of the chitosan nanofiber separator was isotropic.

### 3.3. Thermal Analysis

The excellent thermal stability of the separators is advantageous to prevent internal short circuits of LIBs due to high temperatures caused by overcharging [45]. Therefore, the thermal stability of separators is a critical property in evaluating the safety of LIBs. DSC tests were carried out to measure the thermal stability of the separators. It was found that the chitosan nanofiber separators presented a clear heat absorption peak at 320 °C, which was lower than that of the Celgard separator (470 °C), as shown in Figure 4C. However, as a non-thermoplastic polymer material, chitosan had no heat deformation temperatures, indicating that the chitosan nanofiber separators did not undergo deformation at high temperatures [46]. The thermal dimensional stability of chitosan nanofibers and Celgard separators can be clearly observed through thermal shrinkage experiments treated from 30 to 160 °C for 30 min (Figure 4D). The chitosan nanofiber separators exhibited excellent dimensional stability with no significant deformation after heat treatment at 160 °C; this result is owing to the well-interconnected network structure of the chitosan nanofiber separator [47]. Therefore, the chitosan separator with better dimensional stability is a suitable choice for LIB applications under high temperatures. In comparison, the Celgard separator showed a serious dimensional deformation with a shrinkage rate of 32%. This obvious shrinkage was mainly due to the inherent thermal sensitivity of polyolefins [48].

### 3.4. Wettability

**Figure 5:**
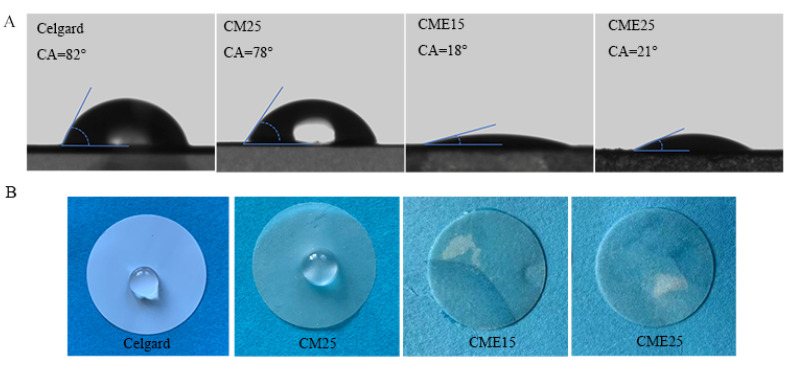
(**A**) Contact angles of chitosan and Celgard separators. (**B**) Wet areas of electrolyte for chitosan and Celgard separators after 30 s.

The affinity between the separators and the polar electrolyte presents a significant influence on the electrochemical properties of LIBs, and good wettability, in turn, facilitates the rapid penetration of the electrolyte inside the separator, which improves the electrochemical performance of the LIBs [49]. According to the test results of the n-butyl alcohol porosimetry, the porosity of the CME15 and CME25 separators was 69% and 54%, respectively, which is much higher than that of the commercial Celgard separator (39%). Undoubtedly, a higher porosity is beneficial to further increase the electrolyte storage capacity of the separator [50]. To further evaluate the electrolyte wettability, contact angle measurements and analysis of liquid electrolyte wetting behavior were carried out (Figure 5). A smaller contact angle confirmed better electrolyte affinity [51]. It can be observed that the electrolyte was able to diffuse rapidly in the CME15 and CME25 separators, with contact angles of 18° and 21°, respectively; whereas, the Celgard separator presented poor wettability with contact angles of 82° due to its intrinsically hydrophobic nature and low surface energy [47]. The better wettability of the CME separators can be ascribed to the improved porosity and the good affinity of the polar functional groups (-NH_2_ and -OH) on the chitosan chain with the polar electrolyte [52,53]. In addition, the electrolyte formed obvious oval droplets on the surface of CM25 with a surface contact angle of 78°. This is because the CM25 separator without ethanol treatment had a dense structure making it hard to be infiltrated. In summary, the CME separators exhibited good electrolyte affinity, which resulted in excellent electrochemical performance in the assembled LIBs.

### 3.5. Electrochemical Performances

**Figure 6:**
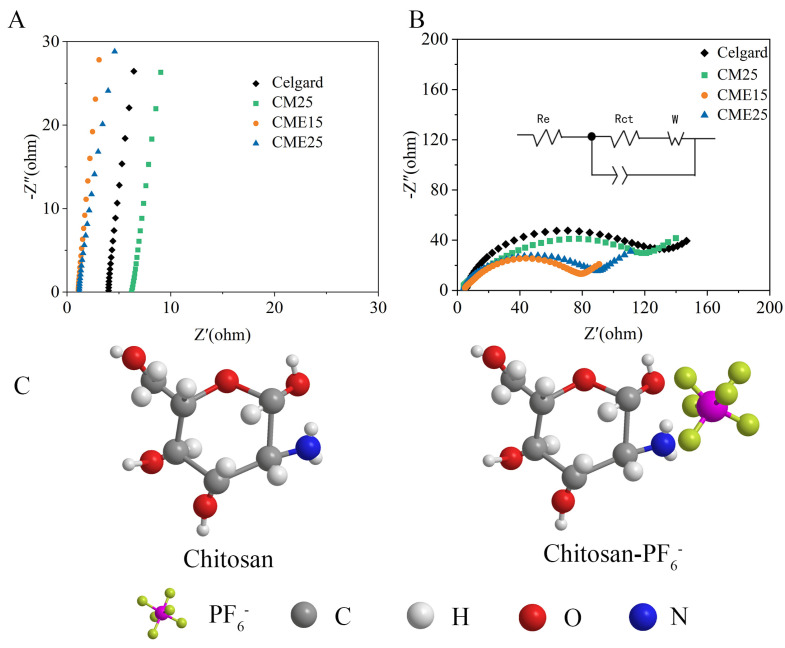
(**A**) Nyquist plot of SS/separator/SS batteries at room temperature. (**B**) Nyquist plot of Li/separator/Li batteries. (**C**) Monomer structure optimization model and complex structure optimization model.

The ionic conductivity of the separator is a crucial property affecting the performance of LIBs, and high ionic conductivity contributes to increasing the energy density of the LIBs. According to the Nyquist curves of the symmetric steel cells (Figure 6A), the x-intercept at high frequency represents the bulk resistance (R_b_). The bulk resistance of CME15 and CME25 was 1.1 and 1.2 Ω, respectively. In contrast, the bulk resistance of Celgard was 3.5 Ω. The corresponding ionic conductivity was calculated using Equation (4) and is summarized in Table 2. The ionic conductivity of porous CEM15 and CEM25 separators was 0.68 mS cm^−1^ and 1.04 mS cm^−1^, respectively, which was higher than that of Celgard (0.36 mS cm^−1^). This is due to the fact that the CME15 and CME25 separators had higher porosity and superior liquid electrolyte wettability, which ensured abundant micropore pathways to allow the transport of ions, thus increasing ionic conductivity. However, the bulk resistance and ionic conductivity of CM25 were 6.1 Ω and 0.21 mS cm^−1^ at the lowest because Li^+^ migration was confined to the dense structure of the separator. A point worth noting is that the impedance of the CEM15 separator was lower than that of the CME25 separator, indicating that as the thickness of the separator decreases, it is beneficial to reduce the impedance value of the separator.

The interfacial impedance indicates the compatibility between the separator and the electrodes, and a lower interfacial resistance facilitates the cycling stability of the LIBs [54]. The compatibility was evaluated using the Nyquist curves in a Li/separator/Li battery (Figure 6B). The interfacial impedance of CM25, CEM15, and CME25 separators was about 119 Ω, 80 Ω, and 90 Ω, respectively, which was lower than that of the Celgard separator (131 Ω). This benefits from the following reasons: (1) the H-bond network structure formed by the amino and hydroxyl groups promoted a high stability of the separator; (2) the amino and hydroxyl groups were highly similar in polar compatibility to the electrolyte, improving the electrolyte affinity. Therefore, the enhanced electrolyte wettability provided more pathways for Li^+^ migration, which weakened ohmic polarization.

**Figure 7:**
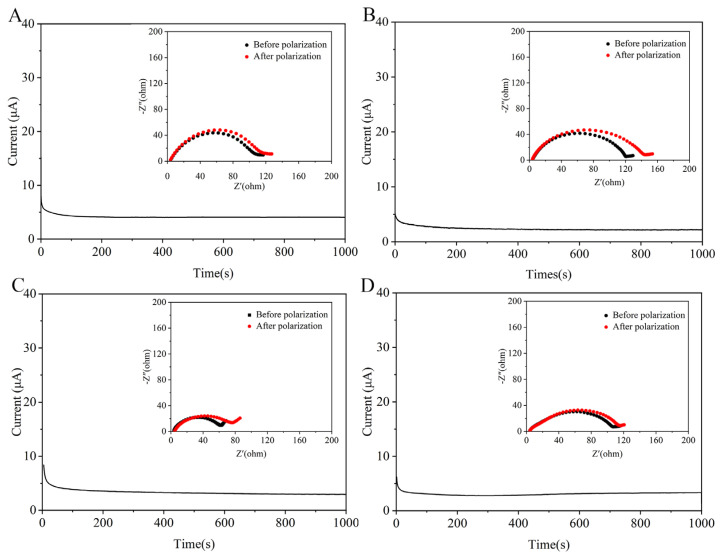
Chronoamperometry of Li/separator/Li batteries under a voltage of 10 mV (inset is the corresponding EIS profiles for the same batteries before and after polarization): (**A**) Celgard, (**B**) CM25, (**C**) CME15, (**D**) CME25.

The Li/separator/Li battery was assembled to investigate the lithium-ion transport number (tLi^+^). A larger tLi^+^ is beneficial to decrease the concentration polarization phenomenon caused by anion enrichment, which suggests a higher Li^+^ migration ability. The tLi^+^ was calculated using Equation (5) and combined chronoamperometry (CA) and EIS methods (Figure 7). The Li^+^ transfer number of Li/CME/Li batteries was 0.5 and 0.49, respectively, and was higher than the Celgard separator (0.26) and CM25 separator (0.2), which demonstrated that the mobility of solvated anions was restricted in the solvent channels of CME separators. This is due to two reasons: (1) the ${\mathrm{PF}}_{6}^{-}$ ions were physically suppressed by the three-dimensional network of CME separators [55]; (2) the electrolyte anions (${\mathrm{PF}}_{6}^{-}$) as a Lewis acid were trapped by the Lewis base (-NH_2_), allowing more Li^+^ to migrate during the charge/discharge process [56]. This further verifies the influence of -NH_2_ on anion transport in the chitosan fiber separators. The density function theory (DFT) was used to calculate the interaction between the -NH_2_ and ${\mathrm{PF}}_{6}^{-}$. The models of chitosan structure optimization were obtained by (Givew6.0) and the binding energy of the composite model with anions in lithium salts was calculated using Guassian16. The calculations showed that the binding energy of -NH_2_ and ${\mathrm{PF}}_{6}^{-}$ was 4.27 eV, which was stronger than that of other natural materials, such as cellulose and chitin [30,36]. This indicates that -NH_2_ limits the migration of anions in liquid electrolyte, thereby allowing relatively easier Li^+^ transport [33].

**Figure 8:**
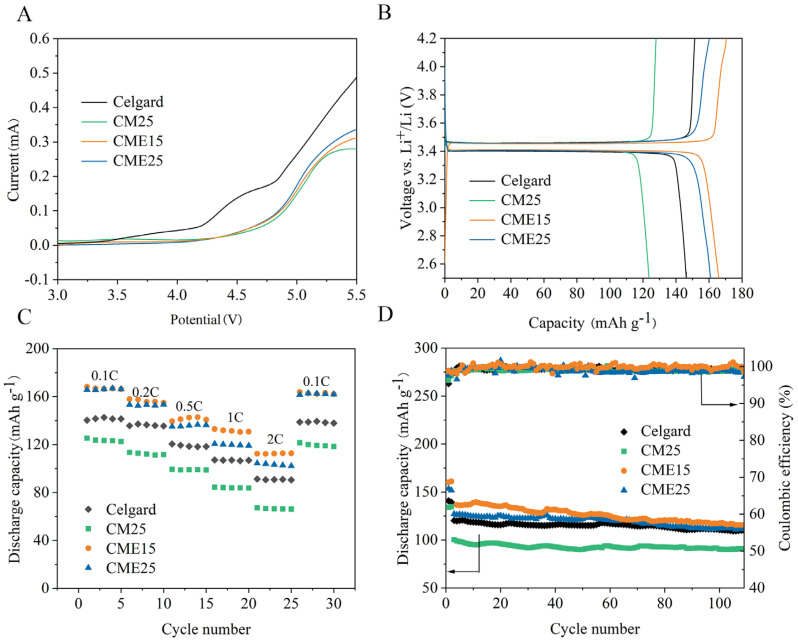
(**A**) LSV of SS/separator/Li batteries at a scan rate of 1.0 mV s^−1^. (**B**) First charge-discharge curves of LiFePO_4_/separator/Li half-batteries at 0.1 C rate (1 C = 170 mAh g^−1^). (**C**) Rate performances of LiFePO_4_/separator/Li batteries. (**D**) Cycling performances of LiFePO_4_/separator/Li batteries at 0.1 C for two activation cycles and 1.0 C for the following cycles.

Separators as inactive components in LIBs should have good electrochemical stability within a wide voltage range. Li/Separator/SS batteries were assembled by LSV to test the electrochemical window of Celgard and chitosan separators (Figure 8A). The voltage of the electrochemical oxidation decomposition signal started from a sudden increase in current [57]. It can be observed that the chitosan separators maintained an essentially stable current at about 4.7 V, whereas the Celgard separator maintained just 4.1 V. Thus, we conclude that the chitosan separators exhibited higher electrochemical stability. First, compared to the Celgard separator with a monolayer pore structure, the chitosan separator with a three-dimensional interconnected porous structure is advantageous to improve electrolyte storage capacity. Second, the Lewis acid (${\mathrm{PF}}_{6}^{-}$) can be assimilated to the Lewis base (-NH_2_), which thus alleviates the oxidative decomposition of anions on the anode [58].

To prove the superiority of the batteries using the CME separators, LiFePO_4_/separator/Li half-batteries were assembled. The initial charge-discharge curves of batteries assembled with Celgard and chitosan separators were tested at 0.1 C with cutoff voltages of 2.5 V to 4.2 V to measure the maximum discharge capacities. The batteries with chitosan separators showed typical voltage plateaus of Fe^2+^/Fe^3+^, as shown in Figure 8B [59]. The initial specific discharge capacity values of CME15 and CME25 were 165 mAh g^−1^ and 161 mAh g^−1^, respectively, which is slightly higher than that of the batteries assembled with a commercial Celgard separator (146 mAh g^−1^). Additionally, the specific discharge capacity of CM25 was just 123 mAh g^−1^, which was consistent with previous experiments.

Figure 8C shows a comparison of the rate properties assembled with different separators (0.1 C to 2.0 C). The discharge capacities of the Celgard and chitosan separators maintained a gradual decrease with increasing current density. At a lower current density of 0.1 C–0.5 C, the discharge capacities of the batteries assembled with CME separators exhibited the equivalent discharge capacities. Specifically, compared with the Celgard separator, the batteries assembled with the CME separators had higher discharge capacity under higher discharge rates (i.e., 1.0 C and 2.0 C). When the current density increased to 2.0 C, the discharge capacity of the batteries using CME15 separators was 112 mAh g^−1^, which was higher than that of the cells assembled with CME25 separators (103 mAh g^−1^). This is owing to the thinner and higher Li^+^ transport number of the CME15 separator, which improved the rate capacities of LIBs. Furthermore, batteries with the Celgard and chitosan separators both returned to the normal discharge capacity level when the current density cycled back to 0.1 C.

The stable cycling performance of charge/discharge capacity is important for LIBs. Batteries with Celgard and chitosan separators were evaluated at a current density of 1 C/1 C (Figure 8D). It was observed that the batteries assembled with Celgard and chitosan separators exhibited almost the same stable Coulombic efficiency after 100 cycles. Compared to the Celgard separator (110 mAh g^−1^), the batteries using CME15 and CME25 separators remained at 117 mAh g^−1^ and 112 mAh g^−1^ after 100 cycles, respectively. This result is owing to better compatibility with electrolytes and a higher Li^+^ transport number of CME separators. However, the discharge capacity of the CM25 separator was just 90 mAh g^−1^ after 100 cycles. This is related to its dense structure, which impeded Li^+^ migration.

**Table 2:** Comparison of relevant electrochemical parameters reported in previous and present work.

Samples	Ionic Conductivity (mS cm^−1^)	Li^+^ Transference Number	Rate Performance (2C, mAh g^−1^)	Battery Performance after 100 Cycles (%)	Reference
PSF	2.93	0.42	120	(0.5C) 95.1%	[60]
OBCS-200	2.9	0.56	116	(0.5C) 83.2%	[36]
PU-PC	1.59	/	115	(0.2C) 95%	[61]
MSD-PI	0.66	0.78	/	(0.2C) 98.3%	[62]
UiO-66-NH2	3.1	/	85	(1C) 81%	[63]
ZIF-67@CNFs	1.55	0.45	125	(0.5C) 88.4%	[64]
CME15	0.68	0.5	112	(1C) 85%	This work

The battery performances of the developed separators and other separators are summarized in Table 2. Compared to other separators, the chitosan separators exhibited similar and, in some cases, better battery performances, which suggests that the chitosan separators have potential to be applied in LIBs. Considering environmental concerns and the circular economy, the chitosan separator can resolve the conflict between sustainability and performance. Additionally, the simple processing process and low cost associated with chitosan separators allow for large-scale production.

## 4. Conclusions

In this work, environmentally friendly and high-performance chitosan nanofiber separators were prepared using a paper-making process, and then they were processed in an ethanol bath. The use of ethanol can effectively improve the porosity of chitosan nanofiber separators. The thickness of the presented separator (CME15) was 15 μm with a density of 0.54 g cm^−3^. The developed CME15 exhibited a high porosity of 69% and electrolyte uptake of 324%. In addition, its superior thermal dimensional stability (without obvious deformation at 160 °C for 30 min) and excellent tensile strength guarantee the safety of LIBs. More importantly, the batteries assembled with the CME15 separator not only exhibited good electrochemical stability (4.7 V) and a high Li^+^ transference number (0.5), but also had good discharge capacity after 100 cycles (117 mAh g^−1^ at 1 C) and excellent C-rate performance (112 mAh g^−1^ at 2 C), demonstrating the potential of CEM separators as high-performance separators for LIBs. This work was expected to provide a new feasible strategy for fabricating a new generation separator.

## Figures and Tables

**Table 1:** Comparison of relevant physical parameters between Celgard and chitosan separators.

Samples	Thickness (μm)	Density (g cm^−3^)	Porosity (%)	Gurley (s)	Tensile Strength (MPa)	Uptake (%)
CM25	25	0.91	34	-	47	98
CME15	15	0.54	69	379	32	324
CME25	25	0.71	54	480	37	267
Celgard	25	0.59	39	549	153/11	124

## Data Availability

Not applicable.

## Supplementary Materials

The following supporting information can be downloaded at: https://www.mdpi.com/article/10.3390/polym15183654/s1, Figure S1: SEM images of Celgard separators. (D) Celgard2325 separator and the corresponding cross-sectional images.
