# Supplementary material for: A Light-Thin Chitosan Nanofiber Separator for High-Performance Lithium-Ion Batteries
Source: Polymers (Basel). 2023 Sep 5;15(18):3654. doi: 10.3390/polym15183654 (PMC10648088; doi:10.3390/polym15183654)
Supplement: Supplementary file 1 [file polymers-15-03654-s001.zip › polymers-2532990-supplementary.pdf]

polymers

Supplementary Materials

A Light-Thin Chitosan Nanofiber Separator for High-Performance Lithium-Ion Batteries

Yanghui Song [1], Guanglei Zhao [1,*], Sihan Zhang [1], Chong Xie [1] and Xiaofeng Li [2,*]

[1] State Key Lab of Pulp and Paper Engineering, School of Light Industry and Engineering, South China University of Technology, Guangzhou 510641, China; syh_hard369@163.com (Y.S.); 201710104778@mail.scut.edu.cn (S.Z.); 201810107072@mail.scut.edu.cn (C.X.)
[2] School of Food Science and Engineering, South China University of Technology, Guangzhou 510644, China
* Correspondence: glzhao@scut.edu.cn (G.Z.); xflibio@scut.edu.cn (X.L.); Tel.: +86-20-87111770 (G.Z.)

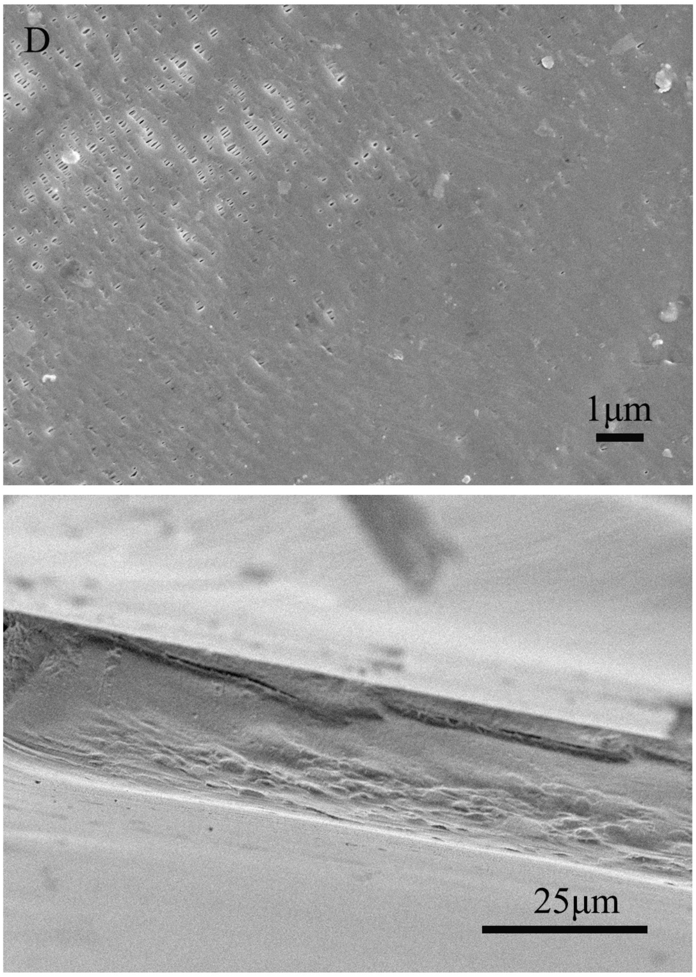

Figure S1. SEM images of various separators. (D) Celgard2325 separator and the corresponding cross-sectional images (d).
